# Radiation Therapy Planning of Thoracic Tumors: A Review of Challenges Associated With Lung Toxicities and Potential Perspectives of Gallium-68 Lung PET/CT Imaging

**DOI:** 10.3389/fmed.2021.723748

**Published:** 2021-08-27

**Authors:** François Lucia, Martin Rehn, Frédérique Blanc-Béguin, Pierre-Yves Le Roux

**Affiliations:** ^1^Radiation Oncology Department, University Hospital, Brest, France; ^2^Service de médecine nucléaire, CHRU de Brest, EA3878 (GETBO), Université de Brest, Brest, France

**Keywords:** radiation—adverse effects, PET perfusion map, radiation planning, lung cancer, stereotactic body radiation therapy, intensity modulated radiation therapy, volumetric modulated arc therapy

## Abstract

Despite the introduction of new radiotherapy techniques, such as intensity modulated radiation therapy or stereotactic body radiation therapy, radiation induced lung injury remains a significant treatment related adverse event of thoracic radiation therapy. Functional lung avoidance radiation therapy is an emerging concept in the treatment of lung disease to better preserve lung function and to reduce pulmonary toxicity. While conventional ventilation/perfusion (V/Q) lung scintigraphy is limited by a relatively low spatial and temporal resolution, the recent advent of ^68^Gallium V/Q lung PET/CT imaging offers a potential to increase the accuracy of lung functional mapping and to better tailor lung radiation therapy plans to the individual's lung function. Lung PET/CT imaging may also improve our understanding of radiation induced lung injury compared to the current anatomical based dose–volume constraints. In this review, recent advances in radiation therapy for the management of primary and secondary lung tumors and in V/Q PET/CT imaging for the assessment of functional lung volumes are reviewed. The new opportunities and challenges arising from the integration of V/Q PET/CT imaging in radiation therapy planning are also discussed.

## Introduction

Radiation therapy has an increasing role in the treatment of both primary and secondary lung cancer (1, 2). In recent years, there has been a technologic revolution in radiation therapy (RT) with the introduction of new techniques, like intensity modulated radiation therapy (IMRT), image guided radiation therapy (IGRT), and stereotactic body radiation therapy (SBRT), which have improved the conformation with the target volumes.

However, radiation induced lung injury (RILI) remains a significant treatment related adverse event of thoracic RT (3). Indeed, the incidence of grade ≥ 2 lung toxicities is about 15-20% (3). Accordingly, a current major challenge of thoracic RT is to better preserve lung function and to reduce pulmonary toxicity.

Radiotherapy for the management of pulmonary lesions is currently based on the so-called anatomical planning. Lung volumes are delineated on CT images, and dose constraints are applied to these anatomical volumes, based on the simplistic assumption that the lungs are functionally homogeneous. However, it is well-established that the distribution of regional function into the lungs is non-uniform, especially in patients with lung cancer who frequently present with tobacco-related lung diseases and cancer treatment induced pulmonary disease.

Functional lung avoidance RT is an emerging concept in the treatment of lung disease.

The technique aim at personalizing RT treatment planning to individual's lung functional distribution, by prioritizing delivery of higher dose in non-functional pulmonary regions while sparing functional areas (4). In that purpose, establishing a functional map of the regional ventilation and perfusion in the lungs is required. Several groups have proposed to use conventional lung ventilation/perfusion scintigraphy and showed a potential interest of functional lung avoidance RT to reduce the dose to the functional lung (4). However, the low spatial and temporal resolution of conventional V/Q scintigraphy has limited accurate mapping of lung functional volumes and restrained its use in daily practice (5).

Ventilation/perfusion (V/Q) positron emission tomography/computed tomography (PET/CT) is a novel imaging modality for regional lung function assessment. As compared with conventional V/Q scan, V/Q PET/CT is inherently a vastly superior technology for image acquisition, with higher sensitivity, higher spatial and temporal resolution, superior quantitative capability, and a greater access to respiratory gated acquisition (5, 6). As a consequence, V/Q PET imaging offers an opportunity to improve the accuracy of lung functional mapping and its use for thoracic radiation therapy planning.

The advent of both advanced radiotherapy techniques and high resolution lung functional mapping is a real opportunity to personalize lung radiation therapy plans to an individual's own lung function and to minimize lung toxicity. Lung PET/CT imaging may also improve our understanding of radiation induced lung injury compared to the current anatomical based dose–volume constraints.

In this review, we will discuss recent developments in radiation therapy for the management of primary and secondary lung tumors, and in V/Q PET/CT imaging for the assessment of functional lung volumes. We will also review the new opportunities and challenges arising from the integration of V/Q PET/CT imaging in radiation therapy planning.

## Current Roles of Thoracic Radiation Therapy

### NSCLC

Lung cancer is the leading cancer in terms of frequency and mortality, with more than 1.6 million deaths per year (7). The role of curative-intent RT is well-recognized in both early stage (8) and locally advanced (9) non-small cell lung cancer (NSCLC).

In recent years, there have been efforts to diagnose lung cancers at earlier and more curable stages using annual low-dose computed tomography (CT) for at-risk populations (10). In the United States, these screening efforts have resulted in up to 30% of lung cancer cases being diagnosed at an early stage, i.e., stage I or II (11). These early tumors are amenable to surgical treatment, with local control rates of up to 96% and overall survival (OS) rates of ~50-60% at 3 years (12). Unfortunately, an important limitation to surgical treatment of these patients is that many have underlying pulmonary or cardiac comorbidities (13). In current practice, RT is the preferred alternative for these patients with early stage (preferably stage I disease with tumor size ≤ 3 cm) NSCLC who are unfit or refuse radical surgery (13).

Approximately 30% of NSCLC patients are diagnosed with locally advanced disease (stage III). This is a heterogeneous group that includes a large number of clinical presentations, often with a significant tumor burden (T3-T4 and N2-N3). Their management requires consultation within a multidisciplinary team. Radical chemoradiation, especially concurrent chemoradiation, and maintenance immune checkpoint-inhibitors (durvalumab) is the standard of care of unresectable stage III NSCLC (14, 15).

### Metastasis

Historically, patients diagnosed with distant metastases secondary to solid tumors were considered incurable and the gold standard of treatment was systemic chemotherapy. Hellman and Weichselbaum suggested that a subset of patients with limited metastatic disease might benefit from aggressive local therapy (16, 17). These patients with a small number (five or fewer) of low volume metastatic lesions were classified as having oligometastatic disease. The management of oligometastatic disease has become a frequent question (18) because increasing evidence has shown that surgical resection or radiation therapy can lead to better outcomes (2). One of the main sites where radiotherapy is used in this setting is in the treatment of lung metastases. Stereotactic body radiation therapy (SBRT) is an excellent therapeutic modality, with control rates of >90% reported in prospective and retrospective series (19–21).

## Modern Radiation Therapy

### 3D-CRT

The 1980s saw the advent of three-dimensional conformal radiotherapy (3D-CRT) with the use of computed tomography (CT) for treatment planning and the replacement of Cerrobend blocking with multi-leaf collimators (MLC). These advances have allowed for the automation of radiation field formation and treatment planning that shapes the fields to the tumor volume. For the past decade, 3D-CRT has been the standard of care to treat unresectable local advanced lung cancer. In recent years there has been an increasing use of intensity-modulated radiation therapy (IMRT) (22). IMRT is a form of 3D-CRT where treatment planning system (TPS) determines non-uniform fluence to attain customized dose distribution, where dose is sculpted to target the tumor while sparing proximal organs at risk (OARs). IMRT is carried out by delivery of multiple beamlets of non-uniform fluence. The calculation of fluence is done by high performance computers using algorithms taking an iterative approach, called inverse planning. The inverse planning starts with desired result and works backwards to achieve best possible beam shape and fluence pattern.

IMRT itself has taken many forms including step-and-shoot that delivers discrete intensity levels and sliding window IMRT that delivers continuous intensity levels. Recently, there has been a growing interest in the rotating gantry IMRT techniques, tomotherapy and volumetric-modulated arc therapy (VMAT), that deliver radiation with MLCs and gantry both in motion. This type of radiation therapy allows the patient to be treated with the full 360° beam angle as the radiation source rotates around the patient during the irradiation. A higher modulation of the beam fluence in the whole arc is obtained thanks to the continuous movement of the leaf and the rotating gantry. The different IMRT techniques are equivalent except for a reduction in treatment time with VMAT (23–25).

However, to ensure full exploitation of this technique, it is necessary to monitor changes in tumor and OAR volume and/or position during treatment with image-guided radiation therapy (IGRT), such as cone-beam CT (CBCT) (26). Indeed, IGRT allows ensuring adequate target coverage while improving treatment outcome (27–30).

Since a secondary analysis of RTOG 0617, a randomized phase III trial comparing 60-74 Gy with concurrent chemotherapy in the treatment of inoperable stage III NSCLC in 544 patients, IMRT has become the gold standard technique (31). Indeed, IMRT was associated with similar survival and tumor control outcomes with a significant decrease in grade ≥ 3 radiation-induced lung fibrosis (RILF) compared with RTC3D (3.5% vs. 7.9) despite larger tumors and higher V5s (lung volume receiving 5 Gy or more) but similar V20s and mean lung doses (MLD) (31).

### SBRT

In the mid-1990s, stereotactic “body” radiotherapy (SBRT), also called stereotactic ablative radiotherapy (SABR), was first introduced by researchers at the Karolinska Institute in Stockholm by applying the principles of cranial stereotactic radiosurgery to extracranial tumor sites, particularly to the lung (32). Then, this technique was developed by several centers in the world (33–35). SBRT consists of delivering very high doses per fraction in a small number of sessions (usually between 3 and 8 fractions over 1-2 weeks) corresponding to a so-called hypofractionated scheme. Thus, radiation oncologists can perform an even more precise and tumor conformal radiation therapy with a rapid dose falloff in the lung parenchyma and adjacent structures leading to a higher biological effective dose (aiming at a BED10 of at least 100 Gy) directed to the tumors and to a more important destruction of the tumor cells (36–38).

A randomized trial comparing SBRT (54 Gy in 3 fractions or 48 Gy in 4 fractions) with conventional (66 Gy in 33 fractions) or moderate hypofractionation (50 Gy in 20 fractions) was conducted by the Trans-Tasmanian Radiation Oncology Group (TROG), known as the CHISEL trial. A total of 101 participants were included (2:1 with *n* = 66 SBRT and *n* = 35 conventional). Results showed superior local control (hazard ratio = 0.32, 95% CI 0.13-0.77, *p* = 0.0077) of the primary disease without an increase in major toxicity (3).

Multiple randomized controlled trials have sought to compare SBRT with surgery (lobar or sublobar resection) for stage I NSCLC, either in an unselected population or in a high-risk population (39–41). Unfortunately, all trials were stopped prematurely due to low inclusion rates, despite multiple adjustments of inclusion criteria to increase patient recruitment. However, a survival analysis including two phase III trials (STARS and ROSEL) by Chang et al. (39) demonstrated similar 3-year recurrence-free survival with SBRT or resection (86 and 80%, respectively, *p* = 0.54). OS was in favor of SBRT [95 vs. 79%, hazard ratio = 0.14, 95% confidence interval (CI) 0.017-1.190, *p* = 0.037]. However, these results should be interpreted with great caution because of the presence of numerous biases, including small patient numbers, unbalance cohorts in these two studies, the risk of type I inference error, and the relatively short follow-up. Based on these data, many international scientific societies and networks now consider SBRT as the best treatment strategy for medically inoperable patients with stage I NSCLC (40, 41).

In light of these results with lung SBRT in NSCLC, studies concerning the possible benefit of SBRT in patients with oligometastatic disease in the lung have been initiated (42–45). Recently, results of ongoing trials using SBRT in oligometastatic disease have been presented. They report improved overall survival and progression-free survival compared to standard therapy, confirming the benefit of local ablative therapy in limited systemic disease (46, 47). Thus, it is very likely that the treatment strategy and prognosis of these patients will change significantly. Indeed, more of them will be future candidates eligible for SBRT for lung metastases before and after multiple lines of immunotherapy and/or targeted agents.

## Radiation Induced Lung Toxicities

Radiation exposure of the lungs is common during a course of therapeutic radiation for thoracic malignancies. Radiation-induced lung injury (RILI) encompasses radiation-induced pneumonitis (RIP), inflammation of the lung which may manifest as a dose-limiting acute or subacute toxicity, and radiation-induced lung fibrosis (RILF), a late effect of lung exposure to radiation. The diagnosis of RIP and RILF is based on clinical presentation and may be supported by associated imaging findings. Various grading scales are used (Tables 1, 2).

**Table 1:** Overview about grading scales for radiation-induced pneumonitis.

**Grading scale**	**Grade 1**	**Grade 2**	**Grade 3**	**Grade 4**	**Grade 5**
CTCAE v5.0	Asymptomatic; clinical or diagnostic observations only; intervention not indicated	Symptomatic; medical intervention indicated; limiting instrumental ADL	Severe symptoms; limiting self-care ADL; oxygen indicated	Life-threatening respiratory compromise; urgent intervention indicated (e.g., tracheotomy or intubation)	Death
RTOG	Asymptomatic or mild symptoms (dry cough); slight radiographic appearances	Moderate symptomatic pneumonitis (severe cough); low grade fever; patchy radiographic appearances	Severe symptomatic pneumonitis; dense radiographic changes	Severe respiratory insufficiency/Continuous O2/Assisted ventilation	Death
LENT-SOMA (EORTC)	Asymptomatic or mild symptoms; slight imaging changes	Moderate symptoms; moderate imaging changes	Severe symptoms; increased density imaging changes	Severe symptoms requiring continuous O2 or assisted ventilation	Death

*CTCAE v5.0, Common terminology criteria for adverse events, version 5.0; RTOG, Radiation Therapy Oncology Group; EORTC, European Organization for Research and Treatment of Cancer; LENT-SOMA, Late effects in normal tissue-subjective objective management analysis*.

**Table 2:** Overview about grading scales for radiation-induced lung fibrosis.

**Grading scale**	**Grade 1**	**Grade 2**	**Grade 3**	**Grade 4**	**Grade 5**
CTCAE v5.0	Radiologic pulmonary fibrosis <25% of lung volume associated with hypoxia	Evidence of pulmonary hypertension; radiographic pulmonary fibrosis 25–50% associated with hypoxia	Severe hypoxia; evidence of right-sided heart failure; radiographic pulmonary fibrosis > 50–75%	Life-threatening consequences (e.g., hemodynamic/pulmonary complications); intubation with ventilatory support indicated; radiographic pulmonary fibrosis > 75% with severe honeycombing	Death
RTOG	Asymptomatic or mild symptoms (dry cough); slight radiographic appearances	Moderate symptomatic fibrosis (severe cough); low grade fever; patchy radiographic appearances	Severe symptomatic fibrosis; dense radiographic changes	Severe respiratory insufficiency/ Continuous O2/ Assisted ventilation	Death
LENT-SOMA (EORTC)	Asymptomatic or mild symptoms; radiological abnormalities;10–25% reduction of respiration volume and/or diffusion capacity	Moderate symptoms; patchy dense abnormalities in imaging;> 25–50% reduction of respiration volume and/or diffusion capacity	Severe symptoms; dense confluent radiographic changes limited to irradation field;> 50–75% reduction of respiration volume and/or diffusion capacity	Severe symptoms requiring continuous O2 or assisted ventilation;dense fibrosis, severe scarring and major retraction of normal lung;> 75% reduction of respiration volume and/or diffusion capacity	Death

*CTCAE v5.0, Common terminology criteria for adverse events, version 5.0; RTOG, Radiation Therapy Oncology Group; EORTC, European Organization for Research and Treatment of Cancer; LENT-SOMA, Late effects in normal tissue-subjective objective management analysis*.

Despite the introduction of new radiotherapy techniques, RILI remains a significant treatment related morbidity of thoracic radiation therapy (3).

The occurrence of grade ≥ 2 RIP in the treatment of lung cancer has gradually decreased from 30–35% with 3D-radiotherapy (48) to 29–32% with IMRT (48). Grade ≥ 3 RIP was seen in about 10-15% with IMRT (49). Grade ≥ 2 and grade ≥3 RILF were seen in about 29 and 15% of patients with IMRT, respectively (50, 51).

Although quality of life after SBRT represents an important issue in assessing the effective impact of this radiation modality on patient management, it has been evaluated in few studies. Indeed, the majority of patients receiving SBRT have poor lung function at the time of diagnosis making them unfit for surgery, so it is crucial to know the effect of SBRT on lung function. The occurrence of RIP grade ≥ 2 and grade ≥ 3 are about 17-20% and 6-7%, respectively. The incidences of grade ≥ 2 and grade ≥ 3 RILF are about 15-20% and 5-6% with SBRT, respectively (3).

Thus, the thorax remains a challenging anatomical site for RT delivery, especially for patients with lung comorbidities, and the reduction of pulmonary side effects (and spare lung function) while achieving reasonable local control and sustaining its curative potential is an ongoing challenge faced by radiation oncologists and interdisciplinary treatment teams (3).

## Conventional (Anatomically Based) Planning

Currently, radiotherapy planning is anatomical for the treatment of lung tumors. Indeed, only CT images are used to define the lung volumes to which dose constraints are applied. To minimize toxicity to the OARs, in particular to the “healthy lung,” treatment planning system (TPS) are used to optimize the beam arrangements in order to respect the planning constraints. TPS allows to evaluate the dose delivered to each voxel in a volume of interest. Among the multiple plan options provided by TPS, the plan with the best therapeutic ratio (maximum tumor control with the least possible complications for normal tissues) is chosen. The dose-volume histogram (DVH) is used as a tool for comparison between plans.

In IMRT, the most important modifiable risk factors for RILF are the radiation dose and anatomical volume of lung irradiated, with lung V20 (volume of lung receiving 20 Gy or more) and mean lung dose (MLD) being validated in early studies. Consistency in lung volumes is important for reporting purposes, with the preferred method being total lung volume minus Gross Tumor Volume (GTV). In one of the most comprehensive analyses, a multi-institutional individual patient data meta-analysis investigated dosimetric predictors for RILF in patients treated with concurrent chemoradiation. This study reported symptomatic RILF in 30% of treated patients, with fatal RILF reported in 2% (52). The most important predictors for RILF included V20, older age, and carboplatin/paclitaxel chemotherapy. V20 as a continuous variable was also validated in a subgroup analysis of RTOG 0617 (31). In this analysis, treatment with IMRT was associated with a significantly lower risk of RILF despite having a higher V5, which had been associated with RILF in prior studies. Thus, the goal for radiation treatment planning is to achieve the lowest V20 and MLD possible, while V5 does not appear to be a critical variable. Commonly cited metrics include a V20 <35% and MLD <20 Gy, but lower doses are often achievable with modern treatment planning strategies. Patients with interstitial lung disease (ILD) should be treated with caution, as higher rates of symptomatic and fatal pneumonitis have been reported in this patient population (53).

In SBRT, there is few data about dose-effect relationship between lung anatomical volumes irradiated and the risk or the grade of toxicities. Saha et al. found that lower lobe tumor location, larger tumor size, PTV, mean lung dose, V20, and V12.5 were significant predictors of symptomatic RILF (54). Tumor size has been found to predict toxicity in previous studies (55–57). The only clinical factor associated with symptomatic RIP found was subclinical interstitial lung disease (45 vs. 1.6%) in the study of Okubo et al. (58). A recent review of 97 studies evaluated clinical and dosimetric predictors of RILI. Unfortunately, no threshold level of “tolerance dose volume” was found. However, the results seemed to show that the risk of symptomatic RILI was relatively low (<10-15%) with an MLD of 8 Gy and a V20 of 10-15% (59).

## Functional Lung Avoidance Planning

Current conventional anatomically-based planning simplistically assumes that the lungs are functionally homogeneous. However, it is well-known that the regional distribution of pulmonary function is heterogeneous, especially in patients with lung cancer, who frequently present with tobacco-related lung diseases such as chronic obstructive pulmonary disease (COPD) or emphysema, and treatment induced pulmonary disease from surgery, radiotherapy, or systemic therapies. In order to decrease pulmonary toxicities and preserve lung function, it has been proposed to personalize radiation therapy treatment planning to individual's lung functional distribution, i.e., to limit as far as possible the dose to the functional lung to the detriment of regions with already impaired lung function (4). Recent advances in radiotherapy techniques, with the use of inverse planning and TPS optimization algorithms, has made possible to add a constraint on a “functional lung” volume.

For that purpose, establishing a functional map of the regional function in the lungs is required. In that respect, the principle underlying Ventilation/Perfusion (V/Q) scintigraphy is very attractive. Indeed, lung scintigraphy allows to assess the regional distribution of ventilation and perfusion in the lungs. Ventilation is imaged after inhalation of inert gases or radiolabeled aerosols that reach terminal bronchioles and alveoli in proportion to regional distribution of ventilation. Perfusion is imaged after intravenous administration of macro-aggregated albumin (MAA) particles, which are trapped in the lung capillaries according to the regional blood flow.

Several studies showed that lung scintigraphy may provide additional information to assist in identifying patients at greater risk of radiation pneumonitis (60–62). Furthermore, studies have demonstrated the potential for lung SPECT imaging to be integrated into treatment planning to improve functional dose metrics (4). Several studies reported a reduction of mean functional volumes and mean lung dose when plans were optimized to spare functional lung (4). However, functional lung avoidance planning using SPECT imaging has not yet been adopted in daily clinical practice. Indeed, no clinical benefit has been clearly established so far (4). Furthermore, there are a wide variety of thresholds used for lung functional volumes delineation. This may be explained by the low spatial and temporal resolution of conventional V/Q scintigraphy, which prevents an accurate and reproducible mapping of lung functional volumes.

## V/Q PET/CT Imaging: A New Imaging Tool for Functional Lung Avoidance Planning

V/Q PET/CT is a novel imaging modality for regional lung function assessment. The same carrier molecules as conventional V/Q scintigraphy are used, but they are labeled with ^68^Gallium, a ß+ isotope, instead of ^99m^Tc, allowing acquisition of images with PET technology (5, 63, 64). Similar physiological processes are therefore imaged using conventional V/Q scan or V/Q PET/CT, but PET is a vastly superior technology for image acquisition, with higher sensitivity, higher spatial and temporal resolution, superior quantitative capability, and a greater access to respiratory gated acquisition (5, 65). The test has already shown promising results in a variety of pulmonary conditions, such as pulmonary embolism diagnosis (63, 66) or pre-surgical assessment of lung cancer patients (67). Similarly, V/Q PET imaging offers an opportunity to improve the accuracy and utility of lung functional mapping for thoracic radiotherapy.

Besides improving the accuracy of lung functional volume delineation, pulmonary PET/CT imaging is also appealing for several reasons (5, 68). The acquisition time is much lower (5 min) than with conventional V/Q scan. V/Q PET imaging is a simple and non-invasive test, with no contraindication or side-effects, especially related to the injection of contrast media (allergy, renal dysfunction). No special procedure such as fasting or diet is required. The effective radiation dose of the scan is low, similar to the dose of conventional V/Q scan (~2-3 mSv for the PET acquisition), PET and CT respiratory-gated acquisition can be readily performed, which may further improve the accuracy of lung functional volumes delineation and improve the co-registration with the CT used for radiotherapy planning. In nuclear medicine facilities that routinely perform V/Q scans with Technegas, and equipped with a ^68^Ga generator, performing V/Q PET/CT does not require additional resources. ^68^Ga is an extremely convenient radiotracer for clinical use. ^68^Ga generators are increasingly available in nuclear medicine facilities owing to growing use for prostate cancer and neuroendocrine tumor imaging.

Le Roux et al. studied, in 30 lung cancer patients, the correlation between ^68^Ga V/Q PET/CT functional volumes and pulmonary function tests (PFTs) indices (69). The results showed that a percentage of lung volume with normal ventilation and perfusion >90% correctly identified impaired lung function in 93% of patients. A high degree of correlation between all functional lung volumes on V/Q PET/CT imaging and lung function as assessed by PFTs was also demonstrated. These results support the possible use of ^68^Ga V/Q PET/CT to predict potential consequences and side effects of treatments that may alter regional function, such as RT.

Although V/Q PET/CT is a very attractive test tool for functional lung avoidance planning, only few studies has been published on the topic so far. A first study simulated a 4D PET/CT perfusion-based radiotherapy plan in a cohort of NSCLC patients (70). These patients were planned to receive curative 3D-CRT at a dose of 60 Gy in 30 fractions. In this study, the definition of “perfused” or “well-perfused” lung on ^68^Ga-MAA PET/CT was based on an automated contour encompassing any ^68^Ga-MAA uptake or a maximum standardized uptake value (SUVmax) cutoff of 30%, respectively. To exclude any areas of clumping or other artifacts, automated contours were manually corrected. This PET/CT-guided planning improved the functional parameters V30, V40, V50, and V60 (all *p*-values < 0.05), and the mean functional dose to the lung was improved by a median value of 0.86 Gy (*p* < 0.01) for well-perfused lungs.

The same team evaluated the value of ventilation in addition to perfusion for functional lung volume sparing in IMRT. Perfusion-guided IMRT planning alone was able to decrease the functional lung dose while maintaining a consistent plan quality (71).

**Figure 1:**
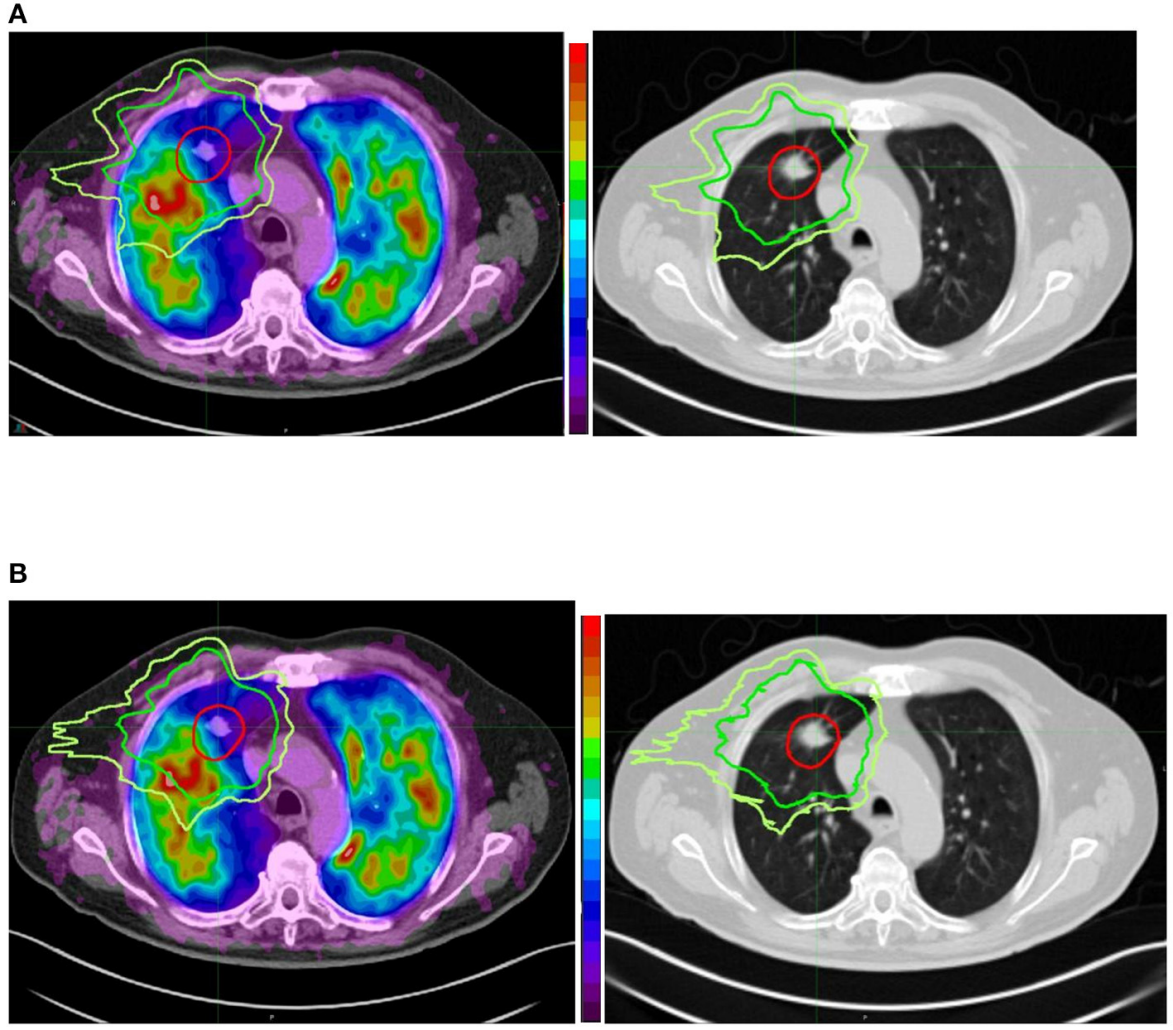
Comparison between conventional (anatomically based) planning **(A)** and functional lung avoidance planning guided by ^68^Ga Q PET/CT **(B)** in a patient treated for a lung tumor by stereotactic body radiation therapy. Both planning show optimal coverage (red isodose line) of the tumor but the functional planning shows a decrease of the dose (20 Gy isodose in green and 16 Gy isodose in yellow) to the functional lung (in red the most functional areas and in blue the least functional areas). We can also see that on the corresponding anatomical images we cannot see any difference between the functional and non-functional areas.

In the same population, changes in perfusion, ventilation, and CT lung density were assessed (72). The authors used deformable registration to register the functional images with RT planning (73). Then, they averaged the isodose volumes in 10 Gy bins intervals. Finally, the relative SUV loss was analyzed for ventilation and perfusion for each dose bin. They showed an almost perfectly linear negative dose-response relationship for perfusion (r2 = 0.99, *P* < 0.01) with a strongly negative correlation for ventilation (r2 = 0.95, *P* < 0.01). In some patients, peritumoral reperfusion/reventilation occurred. These results suggest that the effects of postradiotherapy may be closely correlated with impairments in perfusion, more so than ventilation. Thus, it would be necessary to preserve primarily the function of perfused lungs. In ^68^Ga V/Q PET/CT planning optimization, the aim would be to increase the weight of the radiation beams through the non-perfused lung regions and avoid the perfused lung regions without compromising the plan in terms of satisfying the established criteria for tumor coverage and preservation of other normal tissue (e.g., Figure 1).

## Perspective and Future Challenges

For several years, radiotherapy in lung cancer has seen major technological advances, with the advent of motion management control (4D-CT, respiratory gating), image guidance, intensity modulated volumetric techniques or SBRT, which have led to an improvement in efficacy and tolerance. Nevertheless, further improvements in radiation therapy planning to spare lung function would be of interest for several reasons.

First, most of patients referred for thoracic radiation therapy have poor pulmonary function or have been heavily pretreated. Limiting the risk of high grade/fatal radiation pneumonitis remains therefore a major challenge. Second, multiple subsequent treatments are likely to be necessary for both primary NSCLC (second tumors) and lung metastases (oligo-progression). In these patients, toxicity should be kept as low as possible from the first treatment to preserve the possibility of re-irradiation, especially in SBRT. Third, patients with larger tumors or tumors close to critical structures would probably benefit the most from high-precision SBRT (74, 75), given the absence of other options and the higher risk of toxicity with current technology.

Moreover, V/Q PET/CT could be useful to better assess the relationship between radiation dose and lung toxicity. Indeed, in previous studies, the normal lung volume definitions for dose-volume histogram calculation are highly variable (76, 77). Usually, lung volume was defined as the bilateral lung excluding the planning target volume (Lung-PTV) (78–80) or excluding the gross tumor volume (Lung-GTV) (81–83). However, in RTOG 0617, lung volume was defined as the bilateral lung volume excluding the CTV (Lung-CTV). Currently, both RTOG and ESTRO-ACROP guidelines recommend using Lung-GTV delineation instead of Lung-PTV to standardize lung volume definition among different institutions (84, 85). However, there is only limited clinical evidence on which normal lung definition is better for symptomatic RILF prediction. The definition of a functional lung volume may be of interest to better predict toxicity.

Before implementation of functional lung avoidance planning using lung PET/CT imaging, a number of issues need to be resolved. Firstly, the definition of functional lung volumes was not consistent throughout publications. There is no consensus on the definition of the optimal functional region of the lung. The majority of current planning systems require the definition of a functional threshold, but continuum-based planning is possible (14, 32). Another possibility could be to define multiple levels of functional lung regions that do not overlap, with the most important dosimetric goals assigned to the most functional regions. Secondly, the dose constraints to the functional lung must be clarified. In the two studies that evaluated PET-guided functional lung avoidance planning, the functional lung dose constraints used were the same as those for anatomy-based planning. Most importantly, there is currently no publication that demonstrated the clinical benefit of functional lung imaging over anatomical lung imaging for radiotherapy planning. Indeed, no randomized interventional study has evaluated this question. Thus, it is necessary to test this hypothesis in a phase 3 randomized trial with appropriate statistical power to demonstrate a possible decrease in RILI using functional lung imaging.

Finally, there are currently different options for lung ventilation and perfusion imaging (SPECT, MR, ventilation CT and PET/CT) for the implementation of image-guided functional planning.

The availability and cost of the gas, the expertise required for gas imaging, including access to specialized equipment, and the need for image registration to the planning CT have been perceived as limitations to the clinical implementation of MR hyperpolarized gas imaging techniques in radiotherapy (86–88).

4D CT based methods have also shown promising results to estimate regional ventilation lung function (89, 90). However, high quality CT imaging is a key requirement given the potential for artifacts (90, 91). Furthermore, data in the literature seem to favor the use of perfusion data rather than ventilation for functional optimization of dose delivery to the lung. Indeed, perfusion defects are more frequent than ventilation defects, and both are more frequent than CT changes (92).

Thus, ^68^Ga Q PET/CT seems to be the imaging technique that can provide interesting functional data to guide radiotherapy with a fast and cost-effective imaging.

Two ongoing prospective trials should provide important data to support the initiation of such large scale randomized controlled trials.

Bucknell et al. are investigating a prospective dose escalation study with PET-guided radiotherapy planning that aims to show the value of PET-guided radiotherapy for IMRT stage III NSCLC (93). The HI-FIVE trial is a single arm interventional trial integrating ^68^Ga V/Q PET/CT respiratory-gated (four-dimensional) into radiation treatment planning to identify highly functioning lung volumes and avoidance of these using VMAT planning (NCT03569072). The aim is to evaluate the possibility for moderate dose escalation to the primary tumor only, while respecting conventional normal tissue toxicity constraints. For each patient, his radiation plan will be tailored to the location of his tumor and his lung functional mapping. Feasibility of this study is defined as meeting all dosimetric criteria for ≥15 of 20 patients. Thus, the purpose of this study is to provide valuable information on the feasibility of a larger-scale randomized trial and not to show a possible reduction in pulmonary toxicity or improvement in tumor control.

Our group has recently launched the PEGASUS trial, a single arm monocenter study in patients treated with SBRT for primary or secondary lung lesions (NCT04942275). Initially, patients will receive standard, “anatomical” planning, blinded to the results of the ^68^Ga Q PET/CT. In a second phase, patients will benefit from functional planning guided by ^68^Ga Q PET/CT. Each patient will be his own control. The doses to the functional lung will be calculated and compared for the two treatment planning. The primary aim is to estimate the percentage of patients for whom the dose to the functional lung can be reduced while respecting the standard constraints. Patients will have a clinical follow-up at 1 month and then every 3 months for 1 year. They will also undergo PFTs and repeated ^68^Ga Q PET/CT at 3 months and 1 year to assess the impact of SBRT on global and regional lung function.

## Conclusion

Technological advances in radiation modalities have revolutionized the treatment of primary and secondary lung tumors. The advent of lung PET/CT imaging opens new perspectives for functional lung avoidance planning, in order to improve the therapeutic index, the technique may allow to decrease the dose to the functional lung and to reduce pulmonary toxicity, which may increase the possibility of dose escalation and re-irradiation. Results from ongoing clinical trials should help to guide future research and further push the boundaries of radiation therapy for years to come.

## Author Contributions

FL and MR: clinical experiment in lung cancer and radiation therapy and manuscript writing/editing. FB-B and P-YL: clinical experiment in PET/CT and manuscript writing/editing. All authors contributed to the article and approved the submitted version.

## Conflict of Interest

The authors declare that the research was conducted in the absence of any commercial or financial relationships that could be construed as a potential conflict of interest.

## Publisher's Note

All claims expressed in this article are solely those of the authors and do not necessarily represent those of their affiliated organizations, or those of the publisher, the editors and the reviewers. Any product that may be evaluated in this article, or claim that may be made by its manufacturer, is not guaranteed or endorsed by the publisher.
